# Use of Extracorporeal Membrane Oxygenation in *Pneumocystis* Pneumonia of an Infant with AIDS

**DOI:** 10.1155/2020/8840131

**Published:** 2020-11-18

**Authors:** Grégoire Cane, Arnaud De Boislambert, Charlotte Sgro, Pierre Lavedan, Hélène Foulgoc, Nadir Tafer, Alexandre Ouattara

**Affiliations:** ^1^CHU Bordeaux, Department of Anaesthesia and Critical Care, Magellan Medico-Surgical Centre, F-33000 Bordeaux, France; ^2^Univ. Bordeaux, INSERM, UMR 1034, Biology of Cardiovascular Diseases, F-33600 Pessac, France

## Abstract

*Pneumocystis* pneumonia is a common complication of cellular immunosuppression and may trigger severe pulmonary complications. Rapid onset of acquired immunodeficiency syndrome is possible in infants infected with human immunodeficiency virus (HIV). We report here the case of a 13-week-old girl who was previously healthy presenting with altered immunity and refractory acute respiratory distress syndrome (ARDS) initially attributed to bacterial pneumonia. Venovenous extracorporeal membrane oxygenation (VV-ECMO) was initiated because her condition was poor. An HIV infection was later fortuitously diagnosed after accidental exposure of a nurse to the child's urine. The mother had congenitally transmitted HIV to the child after late (undetected) infection during pregnancy. The lung lesions were finally attributed to *Pneumocystis* pneumonia. We prescribed combined antiretroviral, antibiotic, and steroid therapy aimed at preventing immune reconstitution inflammatory syndrome. VV-ECMO weaning progressed over 30 days to the time of decannulation, rapidly followed by extubation and hospital discharge. The case highlights the fact that rare curable causes of refractory pediatric ARDS should always be investigated early. VV-ECMO should not be excluded as an ARDS treatment for immunocompromised children.

## 1. Introduction

*Pneumocystis* pneumonia is a common opportunistic infection in patients infected with human immunodeficiency virus (HIV) and can cause acute respiratory distress syndrome (ARDS) associated with a high mortality rate (up to 58% even in intensive care units (ICUs)) [1]. Venovenous extracorporeal membrane oxygenation (VV-ECMO) for adults with acquired immunodeficiency syndrome (AIDS) is generally associated with favorable outcomes [2–5]. However, no pediatric case has been described; the utility of VV-ECMO in children is questionable. Pediatric HIV infection can rapidly evolve to AIDS before 2 years of age; *Pneumocystis* pneumonia is not rare in patients with vertically transmitted HIV [6, 7]. We present here the case of a congenitally HIV-infected infant who developed ARDS associated with refractory hypoxemia and *Pneumocystis* pneumonia. VV-ECMO, combined with cotrimoxazole and a corticosteroid, allowed complete respiratory function recovery. The HIV infection was fortuitously diagnosed, suggesting that immunosuppression status had not been adequately screened.

## 2. The Case

**Figure 1:**
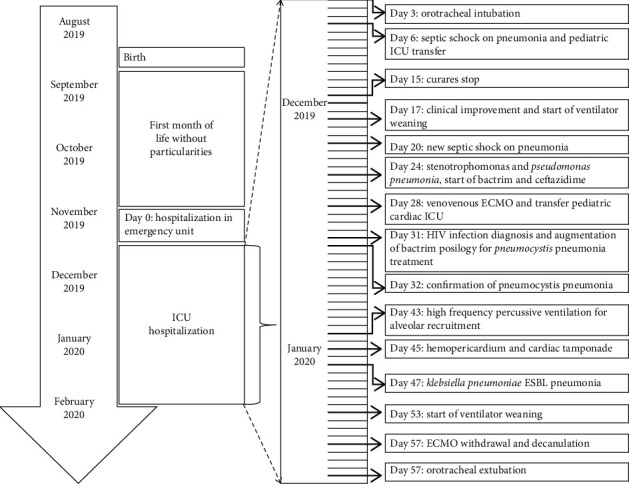
The patient's timeline.

**Figure 2:**
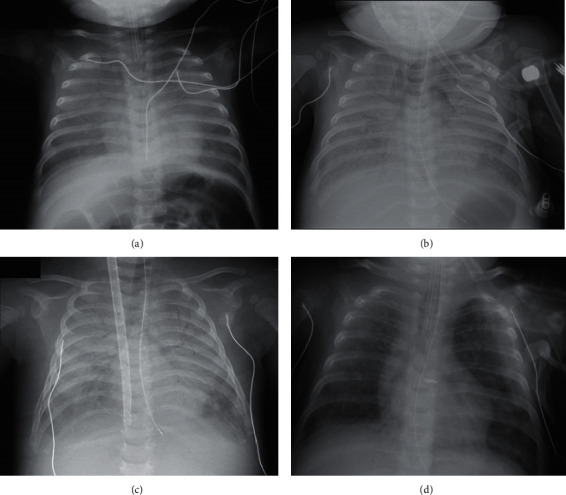
Chest X-rays taken on admission (a): on the day before cannulation and VV-ECMO commencement (b), on the day of VV-ECMO commencement (c), and on the day before extubation (d). Note the Avalon Cannula projecting in front of the superior and inferior vena cava in (c).

At the age of 13 weeks, a girl born at term who had previously been healthy was brought to the emergency unit of a local hospital in respiratory distress (day 0, see Figure 1 for the detailed timeline). The pregnancy had been uneventful; the 6-month maternal HIV screen was negative. The initial clinical evolution was pejorative; the child was placed under mechanical ventilation after orotracheal intubation on day 3. At that time, diffuse alveolo-interstitial lesions were evident on a chest X-ray. Viral PCR revealed a rhinovirus in pulmonary secretions. No other infective agent was noted, but empirical antibiotics were commenced given the gravity of the situation. The condition worsened during a flight between two ICU units, triggering an emergency landing. At arrival in a local, general pediatric ICU, curarization, optimization of ventilation, and prone positioning rapidly improved oxygenation. Such aggressive treatment encouraged the commencement of ventilator weaning on day 17. The favorable clinical course ended on day 20; septic shock developed on ventilation, associated with pneumonia and bacteremia (*Pseudomonas aeruginosa* and *Stenotrophomonas maltophilia*). The antibiotics were changed to ceftazidime and cotrimoxazole. Given the new bacterial infections, possible immunosuppression was evaluated in terms of immunoglobulin and complement component levels; no abnormality was evident. Despite treatment re-escalation and *a priori* antimicrobial therapy, her respiratory status worsened, and uncontrolled hypoxemia developed on day 28. No ventricular dysfunction was evident on transthoracic echocardiography, but major confluent bilateral opacities were apparent on a chest X-ray (Figure 2). Refractory ARDS in the absence of multivisceral failure or right ventricular dysfunction was attributed to bacterial pneumonia, justifying the use of VV-ECMO. After cannulation, the patient was transferred to the cardiac pediatric ICU. VV-ECMO afforded efficient gaseous exchange. Pulmonary compliance was very low at ICU arrival (0.45 mL/cm H_2_O with a tidal volume of 2 mL/kg (i.e., 10 mL); 8 cm H_2_O of positive end-expiratory pressure (PEEP); and 22 cm H_2_O of driving pressure). Antibiotic therapy (cotrimoxazole with 60 mg/kg sulfamethoxazole and 12 mg/kg trimethoprim) and ceftazidime were maintained.

A nurse was accidentally exposed to the child's urine (by pricking herself during collection). In order to decide if a postexposure prophylaxis was indicated, systematic evaluation of viral status of the patient was realized and revealed an HIV-positive serology; the child was found to be HIV-positive on day 31. The viral load as revealed by RT-PCR was extremely high at 6.96 log_10_ copies/mL. The CD4+ lymphocyte count was very low at 72/mm^3^. The mother was diagnosed with an HIV infection that had developed late in pregnancy (after the 6-month screen), which had then been congenitally transmitted to the child. We screened for all classic AIDS co-opportunistic infections; all tests were negative except that for *Pneumocystis carinii*, which was strongly positive on PCR of a tracheal aspirate (8,229 copies/mL). On day 32, the cotrimoxazole dose was changed to 100 mg/kg/day sulfamethoxazole combined with 20 mg/kg/day trimethoprim. Antiretroviral therapy was commenced in association with a corticosteroid (1 mg/kg/day methylprednisolone) to prevent the development of immune reconstitution inflammatory syndrome. Such treatment greatly increased the daily fluid intake (the daily cotrimoxazole alone was delivered in 160 mL of solvent) of the 5 kg infant; we thus commenced VV-ECMO ultrafiltration to control the fluid balance.

**Figure 3:**
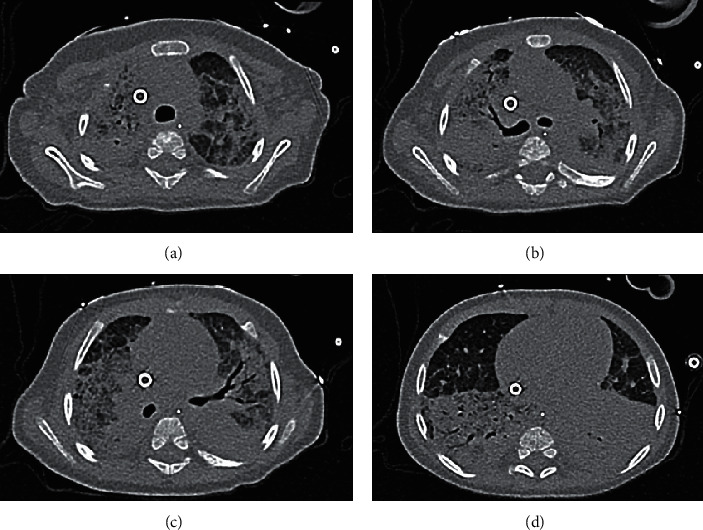
A thoracic computed tomograph taken during VV-ECMO-assisted recovery from *Pneumocystis* pneumonia-caused ARDS. (a) Apical lesions; (b) Carinal lesions associated with fibrosis and alveolar condensation; (c) lower lobe condensation (an air bronchogram); (d) lung base condensations. Note the Avalon Cannula in the vena cava in all panels.

Compliance slowly improved; computed tomography (Figure 3) revealed persistent pulmonary consolidation but without major fibrosis. More aggressive PEEP combined with sessions of percussive ventilation allowed alveolar lung recruitment. At day 45, the hemodynamic status became aggravated. Erosion of the right atrium caused by cannular malpositioning (amplified by the percussive ventilation) had created hemopericardium causing a cardiac tamponade. This was surgically treated; three interventions were required on the same day to iteratively evacuate hemoperitoneum emanating from a lesion in an abdominal arteriole created during the initial procedure. Despite this episode and a new bacterial pneumonia (caused by *Klebsiella pneumoniae* ESBL), her respiratory state progressively improved. Ventilator weaning commenced on day 53 with decannulation on day 57 and extubation and ICU discharge on day 61. The patient was transferred a few days later to a dedicated AIDS center without any need for assisted ventilation. The infant's parents kindly gave us written informed consent for publication of this case report.

## 3. Discussion

To the best of our knowledge, this is the first report of successful ECMO treatment of refractory ARDS associated with *Pneumocystis* pneumonia in an AIDS infant. The Berlin criteria grade ARDS severity with reference to the PaO_2_ : FiO_2_ ratio; severe ARDS is associated with a value less than 100 [8]. Refractory ARDS is a form of severe ARDS that does not respond to mechanical ventilation, prone positioning, or muscle paralysis (to maintain blood saturation) [9]. VV-ECMO is the only method that prevents lung scarring and allows clinical recovery. The Extracorporeal Life Support Organization (ELSO) guidelines for pediatric respiratory failure [10] include a comprehensive list of scenarios in which patients benefit from such exceptional treatment. However, sometimes, ECMO applicability remains unclear. The contraindications [10] include a duration of pre-ECMO mechanical ventilation >14 days, a pre-existing chronic illness, and a poor long-term prognosis. Such conditions are associated with poorer hospital survival of pediatric patients recorded in large ELSO registries [11, 12]. In our case, pre-ECMO mechanical ventilation had been in place for 26 days, and an HIV infection (undiagnosed at the time of cannulation) was present. However, successful decannulation was possible 30 days later. Immunocompromised pediatric patients are at especially high risk of hospital death and are very susceptible to opportunistic infections [12] (survival rates of 33.3 and 57.5% for immunocompromised and nonimmunocompromised infants). However, some pediatric case reports of VV-ECMO used to treat *Pneumocystis* pneumonia reported that patients with hematological diseases and iatrogenic immunosuppression experienced good outcomes [13–15]. Also, the use of ECMO to treat *Pneumocystis* pneumonia was associated with better patient survival than that of patients with other conditions recorded in both the ELSO and Stockholm registries (51 and 89% survival, respectively [16]). The curability of the underlying disease should be considered when deciding whether to commence ECMO. Immunosuppression caused by HIV infection should not prevent the use of VV-ECMO.

Another point of interest is the fortuitous diagnosis of an HIV infection after a nurse had been accidentally exposed to the child's urine. Prior to this discovery, apart from the bacteremia, we lacked clinical, biological, or radiological findings suggestive of immunocompromise. The diffuse alveolo-interstitial lung lesions and the severity of hypo-oxygenation could have been attributable to the initial rhinovirus infection associated with repeated bacterial infections. This case highlights the need to investigate rare but curable causes of ARDS systematically. Inappropriately, neither the ELSO [10] nor the Pediatric Acute Lung Injury Consensus Conference (PALICC) [9] recommended such investigations for pediatric patients with severe ARDS requiring VV-ECMO.

Finally, fluid balance is compromised during intravenous treatment of *Pneumocystis* pneumonia in infants because of the large amount of solvent necessary to deliver cotrimoxazole. The drug is stable for 1 h at the maximum concentration (1/15 dilution; [17, 18]). To deliver 100 mg/kg/day sulfamethoxazole and 20 mg/kg/day trimethoprim using the commercial preparation sulfamethoxazole 400 mg/trimethoprim 80 mg/5 mL, the infant required about 20 mL/kg solvent/day (i.e., 100 mL). Fluid overload should be prevented by prescription of diuretics or via renal replacement therapy (RRT). Continuous RRT is feasible in children weighing up to 10 kg [19], but, in our case, it was easier and safer to link the ECMO and RRT circuits in parallel using the same cannula; this method is well-documented [20].

## 4. Conclusion

We describe here the first case of an infant with AIDS who developed refractory ARDS caused by *Pneumocystis* pneumonia and who was successfully treated via VV-ECMO. Improvement was slow, and severe extracorporeal circulation complications occurred during evolution, but decannulation with a favorable outcome, and ICU discharge, was possible after 30 days of VV-ECMO. AIDS per se should not be a contraindication for VV-ECMO, even in pediatric patients, although evolution will be longer in such patients. The fortuitous diagnosis of HIV infection indicates that systematic biological assessment of rare causes of refractory ARDS must be recommended for pediatric cases. Moreover, our case emphasizes the need to monitor the fluid balance during *Pneumocystis* pneumonia treatment because of the large amount of antibiotic-containing solvent delivered, creating a need for rapid VV-ECMO ultrafiltration to eliminate fluid overload.

## Data Availability

No data were used to support this study.

## Abbreviations

HIV: Human immunodeficiency virus
ARDS: Acute respiratory distress syndrome
ICU: Intensive care unit
VV-ECMO: Venovenous extracorporeal membrane oxygenation
AIDS: Acquired immunodeficiency syndrome
PEEP: Positive end-expiratory pressure
ELSO: Extracorporeal Life Support Organization
PALICC: Pediatric Acute Lung Injury Consensus Conference
RRT: Renal replacement therapy.

## Additional Points

*Summary*. We emphasize that it is important to investigate rare causes of ARDS systematically and highlight the potential benefits of ECMO for such conditions.
